# Misclassification Produced by Rapid-Guessing Identification Methods and Their Suitability Under Various Conditions

**DOI:** 10.1177/00131644261419426

**Published:** 2026-02-23

**Authors:** Santeri Holopainen, Jari Metsämuuronen, Mikko-Jussi Laakso, Janne Kujala

**Affiliations:** 1 University of Turku, Finland

**Keywords:** disengagement, misclassification, rapid-guessing behavior, response time thresholds, simulation

## Abstract

Response Time Threshold Methods (RTTMs) are widely used to identify rapid-guessing behavior (RG) in low-stakes assessments, yet face two key challenges: (a) inevitable misclassifications due to overlapping response time distributions of engaged and disengaged responses, and (b) lack of agreement on which method to use under varying conditions. This simulation study evaluated five RTTMs. Item responses and response times were generated from either a one-component model without RG or a two-component mixture model with RG in the population. Distribution, item, and person parameters were varied. Results showed that when the population contained RG, the mixture lognormal distribution-based method (MLN) was the most robust approach and estimated precise thresholds closest to the time points at which the misclassification rates were minimized, even when bimodality was more difficult to detect. The cumulative proportion method (CUMP) was less robust but also accurate when successful, though less precise. In addition, when the population did not include RG, CUMP was the only method to set thresholds for a notable proportion of cases. The methods were generally more conservative than liberal, though the mixture response time quantile method (MRTQ) was neither. The results are discussed in the light of prior RG research and the methods’ characteristics, and future directions are suggested. Ultimately, for practical settings, we recommend a six-step process for RG identification that utilizes both a mixture modeling approach (MLN or MRTQ) and the CUMP method.

## Introduction

The fundamental assumptions underlying achievement tests are that test-takers will give their best effort, try to actively solve the problems presented in the test items, and provide responses that accurately reflect their knowledge, skills, and abilities (KSAs). In other words, they will exhibit *solution behavior* (SB). However, *test-taker disengagement* violates this assumption because disengaged test-takers may show little to no effort and respond to the test items in ways that do not reflect their true KSAs (Wise, 2017; Wise & Kong, 2005). This phenomenon is especially problematic in low-stakes settings, where there are few or no consequences associated with test performance. In these settings, disengagement is the main construct-irrelevant factor that can negatively impact test score validity (Wise, 2020). In the contemporary digital world, computer-based tests can be administered to measure test-takers’*response times* (RTs). Several studies have demonstrated that RTs can be utilized to detect disengaged responses in addition to actual responses (e.g., Schnipke, 1995; Wise, 2006, 2019; Wise & Kong, 2005). Consequently, differentiating between SB and *rapid-guessing behavior* (RG; or rapid responding [Guo et al., 2024]), in which a test-taker answers an item too quickly to have fully considered it, has been the main challenge in disengagement research for nearly three decades (Wise, 2019).

In disengagement research, two main approaches have been used to differentiate between *rapid guesses* and *engaged responses*. In applied research and operational settings, researchers and test administrators have primarily used *response time threshold methods* (RTTMs) to identify appropriate *time thresholds* at the item level that distinguish between rapid guesses and engaged responses (e.g., Bulut et al., 2023; Guo & Ercikan, 2020; Guo et al., 2016; Holopainen et al., 2025; Lee & Jia, 2014; Rios & Guo, 2020; Schnipke, 1995; Schnipke & Scrams, 1997; Wise, 2019; Wise et al., 2004; Wise & Kong, 2005; Wise & Kuhfeld, 2021; Wise & Ma, 2012). For a brief introduction to these methods, see, for example, Holopainen et al. (2025).

All RTTMs assume that the effort test-takers exhibit and the time they spend answering test items are positively related. Thus, there should be distinguishable differences in RTs under RG and SB. This has led to the idea that when RG is present, the overall item-specific response time distributions are mixtures of rapid guesses and engaged responses, and that there is a region in the distributions where an appropriate time threshold can be found to differentiate between these responses. Using the identified time threshold, item responses are classified as RG or SB based on a binary engagement indicator. This indicator takes a value of 1 if a test-taker’s RT to an item exceeds the time threshold (engaged response) and a value of 0 if the RT is less than or equal to the time threshold (rapid guess) (Wise & Kong, 2005). This engagement indicator can be used in various ways. For instance, one can calculate an overall measure of test-taker effort (e.g., response time effort [RTE]; Wise & Kong, 2005), a measure of effort applied to test items (e.g., response time fidelity [RTF]; Wise, 2006), or filter out rapid guesses to continue data analysis with the filtered dataset (e.g., Guo et al., 2016; Wise & DeMars, 2006; Wise & Kong, 2005).

Another approach to distinguishing rapid guesses from engaged responses is to incorporate engagement state as a latent class variable into item response theory (IRT) models, allowing it to vary across and within test-takers (e.g., Meng et al., 2015; Ulitzsch et al., 2020; Wang & Xu, 2015). For an overview of these models, see Nagy and Ulitzsch (2022). More recently, Liang et al. (2025) proposed a mixture hierarchical model for RG identification that models response correctness as a function of the test-taker’s mastery of relevant attributes. In the current study, however, we focused on RTTMs because the rationale was to address two major challenges of these methods, which we discuss next.

## Study Rationale: Two Major Challenges in RG Identification

RTTMs have several positive features that make them the most popular approach for identifying RG in applied research and operational settings (Rios & Deng, 2021; Ulitzsch et al., 2023). First, RT information from item responses can be automatically collected during a test session without interfering with respondent behavior. Second, RTTMs can identify RG at the item response level, allowing researchers to study RG and/or SB patterns within a test (e.g., Pastor et al., 2019) or item-level correlates of RG (e.g., Wise, 2006) or filter out individual rapid guesses instead of applying listwise deletion to test-takers deemed disengaged. Third, the validity of the RTE score proposed by Wise and Kong (2005) as a measure of test-taker effort has been well-documented (see Wise, 2015). Finally, RTTMs are generally simple to implement in any data analysis software and do not require extensive statistical knowledge. However, although they are useful, using them to identify RG presents two major challenges.

The first challenge is that RT is only a proxy for RG in these methods, and we have a binary conception of test-taking engagement as a psychological phenomenon when responses are classified as either rapid guesses or engaged responses (Holopainen et al., 2025). Since RT distributions of rapid guesses and engaged responses tend to overlap, using time thresholds as binary cutoffs between the two inevitably leads to misclassifications. Researchers have acknowledged that RG may never be perfectly identified (e.g., Ulitzsch et al., 2023). For instance, Holopainen et al. (2025) used choice reaction time as a ground truth variable in RG identification and found that the various RTTMs they used resulted in substantial misclassification rates ranging from 8.0% to 16.4%. Furthermore, disengagement may be a more complex phenomenon than these naive, threshold-based methods can capture. Wise (2017) proposed three potential conditions under which rapid guessing occurs: time running out in high-stakes tests, low motivation in low-stakes tests, and test-takers who glance at the item, quickly recognize they cannot answer it, and then opt to rapidly guess. Moreover, Wise and Kuhfeld (2021) theorized that disengagement can manifest in three ways: rapid guessing, semi-rapid guessing, and partial engagement. In summary, it is important to acknowledge this challenge because especially misclassification of rapid guesses as engaged responses (false negatives), to which the various RTTMs are prone (Holopainen et al., 2025; Rios & Deng, 2021; Wise, 2017), can lead to bias in item and ability parameter estimates (Rios, 2022a, 2022b; Rios & Deng, 2024) and score estimates (Wise & Kuhfeld, 2021).

Second, although identifying RG using RTTMs is the most popular approach, previous research does not appear to agree on the best method for different situations. For example, Soland et al. (2021) compared various RTTMs and discovered inconsistencies in their thresholds. They also found that the performance of these methods depended on the criterion used, such as the number of thresholds identified or the degree to which the accuracy of the identified rapid guesses resembled randomness. In addition, Rios and Deng (2021) conducted a meta-analysis of studies comparing at least two RTTMs. They found non-negligible variation in the proportion of rapid guesses identified by the methods. Overall, disengagement researchers and practitioners would benefit from unbiased information on the validity and usability of RTTMs in different situations, as this would facilitate the selection of an appropriate method.

It should be noted that, based on comparisons of the misclassification rates produced by different RTTMs, Holopainen et al. (2025) recommended a hybrid approach similar to the one used by Rios and Guo (2020). In this approach, a mixture model-based method, such as the mixed lognormal distribution-based method proposed by Rios and Guo (2020), is first employed. If that fails, the cumulative proportion method proposed by Guo et al. (2016) is used. However, Holopainen et al.’s (2025) study was limited to an empirical setting in which the authors used participants’ choice reaction times as ground truths for rapid guessing. Although choice reaction time was a useful way to assess misclassifications, it was only partially a ground truth. The authors noted that their approach likely detected only the totally thoughtless type of rapid guessing from unmotivated test-takers, but rapid guesses from test-takers who quickly recognized they did not know the answer and chose to guess rapidly remained undetected. Furthermore, the participant-specific choice reaction time estimates were uncertain because they were estimated as the participants’ mean log RTs in a separate task. In addition, Holopainen et al. (2025) examined two-choice items only, which hindered the generalizability of the results.

## Research Questions

We aim to address the two major challenges of RTTMs mentioned above: (1) the inevitability of misclassifications, and (2) the absence of agreement on the best method to use under varying conditions. Our approach is to conduct a simulation study in which we can manipulate several model parameters that may affect the misclassification rate produced by the selected RTTMs used to identify RG. We hypothesize that the simulation study results will indicate the conditions under which specific RTTMs succeed or fail to find appropriate time thresholds and ultimately demonstrate the suitability of specific RTTMs under different conditions that may be present in real assessment data. Our research questions (RQs) are as follows:

- **Research Question 1 (RQ1):** How well do the selected RTTMs find the optimal thresholds (i.e., the time points at which the misclassification rates are minimized) when RG is present, or avoid estimating thresholds when no RG is present?
- **Research Question 2 (RQ2):** Which simulation parameters affect the results of which RTTMs, and how? That is, in what conditions do the RTTMs succeed or fail?
- **Research Question 3 (RQ3):** What is the rate and nature of the misclassifications (i.e., the balance between false positives and false negatives) for the RTTMs when RG is present?

In RQ2, success means that a valid threshold was estimated when rapid guessing was present, or no threshold was estimated when rapid guessing was not present. In contrast, failure means that the estimated threshold was invalid or no threshold was estimated when rapid guessing was present, or that a threshold was estimated when rapid guessing was not present. Note that in theory, such engaged responses might have been generated for which the RT indicated a rapid guess, even though RG was not assumed to be present in the population. This prompts the question of whether it was truly a failure if such a small threshold was estimated below which all RTs must have been given by rapid guesses. This fallacy is discussed more thoroughly when we present the limitations of the study.

## Methods

### Data Generation

#### General Selections

We examined two separate situations in the population of item-specific responses and RTs: In the first case, the population was assumed to contain RG, and the data were generated according to a two-component mixture model for responses and RTs, with separate mixture components for rapid guesses and engaged responses. The data were generated separately for the two components using a three-step process. In the second case, we assumed that the population did not contain RG, and hence the data were generated from a one-component distribution. The data were generated identically to the component of the engaged responses in the situation with RG. Therefore, below, when we talk about the SB component (or the engaged distribution) or its parameters, we refer to the engaged component of the two-component mixture model and its parameters when RG is present, as well as the one-component distribution and its parameters when no RG is present.

To examine the sensitivity of the selected RTTMs to fluctuations in sample size, we set the sample size to either 500 or 2,500. The former is a relatively small sample size in large-scale assessments, while the latter is a sample size often seen in these settings (see Rios & Deng, 2024). For the conditions with RG, we set the mixture weight of the RG component (*λ*_*RG*_) to either 0.1 or 0.05. The former represents the average proportion of RG responses found in low-stakes assessments (see Rios et al., 2022), while the latter represents a situation in which rapid guesses may be more difficult to detect, particularly in small samples, as the number of rapid guesses is low. For example, with a sample size of 500, there would be 25 rapid guesses (= 0.05 × 500).

#### First Step: Sampling Person Parameters

Following the general IRT framework for response engagement (see Nagy & Ulitzsch, 2022), we assumed that the probability of making a correct rapid guess was independent of ability and that the rapid-guessing RT distribution was unaffected by person and item parameters (e.g., Meng et al., 2015; Schnipke & Scrams, 1997; Ulitzsch et al., 2020; Wang & Xu, 2015). Therefore, we did not sample person parameters for the RG component. For the SB component, however, we assumed a correlation structure between ability and speed (e.g., Meng et al., 2015; van der Linden, 2007; Wang & Xu, 2015), and sampled the ability (*θ*_*i*_) and speed (*τ*_*i*_) parameter values from $N(\mu_{p},\Sigma_{p})$ , where

(1) $$\mu_{p}={\left(\mu_{\theta },\mu_{\tau }\right)}^{T}={\left(0,0\right)}^{T},$$

and

(2) $$\Sigma_{p}=\left(\begin{array}{c} \sigma_{\theta }^{2}\sigma_{\theta \tau }\\ \sigma_{\theta \tau }\sigma_{\tau }^{2} \end{array}\right)=\left(\begin{array}{c} 1\sigma_{\theta \tau }\\ \sigma_{\theta \tau }0.05 \end{array}\right).$$

The means and variances of *θ* and *τ* are the same as those used by Ulitzsch et al. (2020) in their simulation study. To account for correlations of different magnitudes between ability and speed, we set $\rho_{\theta \tau }\in \left\{0.2,0.5,0.8\right\}$ , where the positive signs imply that more proficient test-takers are faster than less proficient ones. These constraints meant that $\sigma_{\theta \tau }\in \sqrt{1\times 0.05}\times \left\{0.2,0.5,0.8\right\}=\sqrt{\frac{1}{20}}\times \left\{0.2,0.5,0.8\right\}\approx \{0.045,0.112,0.179\}$ .

In the following sections, we make three local independence assumptions consistent with the assumptions in, e.g., van der Linden (2007), Wang and Xu (2015), and Ulitzsch et al. (2020): (1) the responses on an item are conditionally independent given a person’s ability and behavior, (2) the RTs on an item are conditionally independent given a person’s speed and behavior, and (3) the responses and RTs on an item are conditionally independent given the person parameters and behavior.

#### Second Step: Generating Item Responses

Regarding the rapid guesses, we assumed three different situations that could occur in real assessment settings: (1) rapid guessers truly respond randomly, that is, the rapid-guessing accuracy equals the chance level (*g*, e.g., Ulitzsch et al., 2020; Wang & Xu, 2015), (2) rapid guessers respond with lower accuracy than the chance level, and (3) rapid guessers respond with higher accuracy than the chance level. The latter two situations may be due to cognitive biases that favor one option over another, resulting in rapid-guessing accuracy that does not match the chance level. For example, Wise and Kuhfeld (2020) found that the middle options (B and C) in their study of 15 four-choice items (chance level = 0.25) were chosen more often by rapid guessers than the other options (A and D). Option B, for instance, was selected by rapid guessers 30–40% of the time, while option D was selected only 10–20% of the time. Due to this phenomenon, when option B was correct, rapid guessers answered correctly 33% of the time. However, when option D was correct, rapid guessers answered correctly only 14% of the time. In addition, Holopainen et al. (2025) observed a similar phenomenon with two-choice yes-no items, where the group-wise chance levels (based on participants’ language and items’ type) varied between 38% and 62% instead of being close to 50%. Therefore, in this study, when converting rapid-guessing probabilities to responses, we considered all three possible situations; rapid guesses were assumed to be correct if a random number drawn from U(0,1) was less than *g* (situation 1) or less than $g\pm e_{g}$ (situations 2 and 3), where *e*_*g*_ is the error. Otherwise, rapid guesses were assumed to be incorrect. We arbitrarily selected *e*_*g*_ to equal 0.05. Moreover, we assumed two- and four-choice items, and thus g∈{1/4,1/2}={0.25,0.5} .

For the SB component, we generated the engaged response probabilities according to the unidimensional three-parameter logistic (3PL) response model (e.g., Wang & Xu, 2015), that is,

(3) $$p_{i}\left(\theta_{i}\right)=c+(1-c)\frac{\exp\left(a(\theta_{i}-b)\right)}{1+\exp\left(a(\theta_{i}-b)\right)},$$

where *θ*_*i*_ is the ability parameter of person *i*, and *a*, *b*, and *c* are the item discrimination, difficulty, and pseudo-guessing (lower asymptote) parameters, respectively. To account for the discrimination, difficulty, and lower asymptote of different types of items, we set a∈{0.5,1,1.5} , b∈{−2,−1,0,1,2} , and c∈{0,g} . The engaged responses were correct if a random number drawn from U(0,1) was less than $p_{i}\left(\theta_{i}\right)$ and incorrect otherwise.

#### Third Step: Generating RTs

We assumed above that the rapid-guessing RT distribution is unaffected by person and item parameters. Therefore, following Schnipke and Scrams (1997), Ulitzsch et al. (2020), and Wang and Xu (2015), the rapid-guessing log RTs were sampled from

(4) $$N\left(\mu_{\mathrm{RG}},\sigma_{\mathrm{RG}}^{2}\right).$$

In contrast, we applied the widely used lognormal linear model to the SB component for RTs, incorporating person and item parameters (e.g., Klein Entink et al., 2009; Meng et al., 2015; Ulitzsch et al., 2020; van der Linden, 2007; Wang & Xu, 2015). Therefore, following Wang and Xu (2015), each engaged log RT was sampled from

(5) $$N\left(\alpha -\tau_{i},\beta^{2}\right),$$

where *α* and *β* are the time intensity and speed-discrimination parameters, respectively. Let $f_{L|\mathrm{SB}}\left(l;\tau ,\alpha ,\beta^{2}\right)$ denote the conditional density function of log RT given that the participant was in the engaged state, where *L* represents the random variable for log RT and *l* its realized value. Moreover, let $f_{\tau }\left(\tau ;\;\mu_{\tau },\sigma_{\tau }^{2}\right)$ denote the density function of *τ*. By definition, both are normal densities. Therefore, we obtain the marginal density function of log RT in the SB component for a population of test-takers by marginalizing over the underlying distribution of *τ* as follows:

(6) $$\begin{array}{c} f_{\mathrm{SB}}\left(l\right)=\int_{-\infty }^{\infty }f_{L|\mathrm{SB}}\left(l;\tau ,\alpha ,\beta^{2}\right)f_{\tau }\left(\tau ;\mu_{\tau },\sigma_{\tau }^{2}\right)d\tau \\ =f\left(l;\alpha -\mu_{\tau },\beta^{2}+\sigma_{\tau }^{2}\right), \end{array}$$

where $\mu_{\mathrm{SB}}=\alpha -\mu_{\tau }=\alpha$ and $\sigma_{\mathrm{SB}}^{2}=\beta^{2}+\sigma_{\tau }^{2}=\beta^{2}+0.05$ .

In terms of the bimodality of the entire RT distribution in the two-component mixture model, we considered two scenarios and set the location of the rapid-guessing log RT distribution to *μ*_*RG*_= 4.5 or *μ*_*RG*_= 6.5, and the location of the engaged log RT distribution to $\mu_{\mathrm{SB}}=\alpha =7.5$ . We estimated the former rapid-guessing location parameter and the engaged location parameter from real assessment data with clear bimodal patterns in item-specific RT distributions (discussed more thoroughly in the “Data Analysis” section). The latter rapid-guessing location parameter was included to represent situations in which the underlying distribution is bimodal, yet the bimodality is difficult to detect in the observed data. Furthermore, since exp(4.5)≈90 (ms) and exp(6.5)≈665 (ms), cases with *μ*_*RG*_= 4.5 represent the totally thoughtless, rapid guessing, while cases with *μ*_*RG*_= 6.5 may represent rapid guesses from those test-takers who quickly glance at the item and then decide to make a rapid guess. Finally, we set the scales of the rapid-guessing and engaged log RT distributions to *σ*_*RG*_= 1.5 and $\sigma_{\mathrm{SB}}=\sqrt{\beta^{2}+0.05}=0.5$ , respectively, where the latter constraint meant that $\beta =\sqrt{0.5^{2}-0.05}=\sqrt{0.2}\approx 0.447$ . These scales were estimated from the aforementioned real data.

#### Number of Simulation Conditions

In the conditions with RG in the population, the number of simulation conditions was 4,320, and in the conditions without RG, the number of conditions was 360. Therefore, the total number of conditions was 4,680. We replicated each simulation condition 100 times. We gathered all simulation parameters in Table 1.

**Table 1.** Parameters for the Simulation Study.

Parameter	Conditions with RG	Conditions without RG
General parameters		
Number of simulation conditions	4,320	360
Number of mixture components	2	1
Sample size (*n*)	500; 2,500	500; 2,500
RG component’s weight, i.e., the RG proportion (*λ*_*RG*_)	0.05, 0.10	0
Person parameters		
Mean of the latent ability (*µ*_*θ*_)	0	0
Variance of latent ability (*σ*_*θ*_^2^)	1	1
Mean of the latent speed (*µ*_*τ*_)	0	0
Variance of the latent speed (*σ*_*τ*_^2^)	0.05	0.05
Correlation between ability and speed (*ρ*_*θτ*_)	0.2, 0.5, 0.8	0.2, 0.5, 0.8
Parameters of the log RT distribution		
Mean of the RG component’s log RT distribution (*µ*_*RG*_)	4.5, 6.5	-
Scale of the RG component’s log RT distribution (*σ*_*RG*_)	1.5	-
Time intensity parameter (*α*)	7.5	7.5
Time discrimination (*β*)	$\sqrt{0.2}$	$\sqrt{0.2}$
Parameters of the probability of answering correctly		
Chance level (*g*)	0.25, 0.5	0.25, 0.5
Error in the observed RG accuracy (*e*_*g*_)	-0.05, 0, 0.05	-
Item discrimination (*a*)	0.5, 1, 1.5	0.5, 1, 1.5
Item difficulty (*b*)	-2, -1, 0, 1, 2	-2, -1, 0, 1, 2
Pseudo-guessing, i.e., lower asymptote (*c*)	0, *g* (for each *g*)	0, *g* (for each *g*)

*Note*. *RG* = rapid-guessing behavior; *RT* = response time.

### Data Analysis

#### Estimation of the Parameters Used in Generating Log RTs

The parameter values (*μ*_*RG*_= 4.5, *σ*_*RG*_= 1.5, *α*= 7.5, and $\beta =\sqrt{0.2}$ ) used to generate the log RTs were selected based on estimates from a real, large-scale assessment data. A standard two-component mixture model was fitted to the item-specific log RT data, and the means of the estimated parameter values were then calculated across items for both components in the distribution. The data were collected from 6,664 students who completed the d-Lexize test, which assesses vocabulary knowledge (Bertram et al., 2025). The original RT scale was in milliseconds. A more detailed description of the data is provided in Holopainen et al. (2025), who used some of the same RTTMs as in this study to identify thresholds for the d-Lexize items.

#### Response Time Thresholds

The RTTMs applicable to our simulation setting included the *Cumulative Proportion* method (CUMP; Guo et al., 2016) and the *Automatized Visual Inspection* method (AutoVI; Holopainen et al., 2025), as well as three *Mixture Modeling Approaches* (MMAs): the *Mixture Response Time Quantile* method (MRTQ; Holopainen et al., 2025), the *Mixed LogNormal distribution-based* method (MLN; Rios & Guo, 2020), and a modified version of MLN (mMLN), which uses the estimated density of log RT instead of RT density. These methods were applicable mainly because they could be automated during the simulations without manually setting a threshold for each generated dataset.

Before estimating the AutoVI and the mixture model-based thresholds, we assessed the number of mixture components by comparing the fits of a one- and a two-component model to the data based on the Bayesian Information Criterion (BIC; Schwarz, 1978). We used the R package mixtools (Benaglia et al., 2009) to fit the two-component models to the data. If the two-component model fit the data better (lower BIC), the threshold estimation proceeded for AutoVI and the MMAs. When RG was present in the population, 99.8% of the distributions were correctly judged to have two components. In contrast, when RG was not present, 99.9% of the distributions were correctly judged to have one component. A detailed description of applying these methods to the simulated data and justifications for excluding certain methods from the present study are presented in the supplementary material (available at: https://doi.org/10.17605/OSF.IO/VJNR7).

When the sample size increases, more and more complex models fit the data seemingly well. Therefore, in our initial analysis, we also checked whether a three-component model fit the data better than a two-component model for samples of 2,500 log RTs generated based on the two-component model using the parameter values $\lambda_{\mathrm{RG}}\in \{0.05,0.1\}$ , $\mu_{\mathrm{RG}}\in \{4.5,6.5\}$ , and $\rho_{\theta \tau }\in \left\{0.2,0.5,0.8\right\}$ (2 × 2 × 3 = 12 conditions in total). Based on BIC, the three-component model did not fit the data better than the two-component model at any point across 100 repetitions for any of the 12 conditions. Thus, in the actual simulations, we did not test whether a three-component model would have fit the data better than a two-component model, because we expected these results to be replicated.

Before the main analysis, we found that although a two-component mixture model could be estimated in most cases with RG in the population, the RT mode corresponding to the RG component remained hidden in the estimated RT density function for 1.8% of all cases. Similarly, the log RT mode remained hidden in the estimated log RT density function for 47.7% of all cases. Consequently, defining a threshold based on MLN for the 1.8% and based on mMLN for the 47.7% was unjustified, because these methods assume that the estimated density function has a minimum on an open interval between the modes. The high percentage for mMLN suggests that, after fitting a two-component mixture model to the log RT data, examining the estimated density of the original RT rather than the log-transformed RT is necessary for the MLN approach to be meaningful. A figure in the supplementary material (available at: https://doi.org/10.17605/OSF.IO/VJNR7) illustrates this phenomenon. In the main analyses, we treated these cases as missing values.

#### Main Analysis

We used the R 4.5.0 (R Core Team, 2025) software for data generation and analysis. First, to understand the behavior of the methods at a general level and address RQ1, we analyzed the data as follows: In conditions where RG was present in the population, we examined the thresholds across all instances (i.e., all simulation conditions [4,320] and repetitions [100]). More precisely, we examined the distributions of the differences between the estimated thresholds and the time points at which the misclassification rates were minimized in the generated samples, which we refer to as the “optimal” thresholds. Note that in the realized level, there might be more than one time point at which the misclassification rate is minimized. In this study, 69.9% of the generated samples contained one optimal threshold, 21.4% contained two optimal thresholds, 6.2% contained three optimal thresholds, and 2.5% contained more than three optimal thresholds. In the cases with more than one optimal threshold, the average (median) number of observations between the smallest and largest optimal thresholds was 1 (two optimal thresholds), 5 (three optimal thresholds), and 9 (more than three optimal thresholds). In addition, the average differences between the smallest and largest optimal thresholds were 60, 92, and 109 milliseconds, respectively. For each method, we report the results with respect to the optimal thresholds that were closest to the estimated thresholds.

We visualized the distributions of the differences as histograms. In addition, we calculated the means, standard deviations, and biases of the estimated thresholds, that is, the average differences between the estimated and optimal thresholds. In conditions without RG, however, we calculated the proportions of cases for which a threshold was acquired across all instances (i.e., all simulation conditions [360] and repetitions [100]). The smaller the proportion, the better, because a threshold should not have been estimated when RG was not present.

Second, to understand which simulation parameters affected which methods and address RQ2, we analyzed the data as follows: In the conditions with RG, we examined the proportions of specific conditions in specific parts of the distributions of differences from the optimal thresholds (e.g., negative differences vs. positive differences, abnormally large differences, and missing values) based on the information gained from the analysis of the general-level results. In addition, we examined how much variance was explained by accounting for the different parameters. We also visualized the distributions of the differences under certain simulation conditions. In general, regarding the conditions with RG, we were interested in the accuracy, precision, and robustness of the estimated thresholds. By “accuracy,” we mean how close the estimated thresholds were to the optimal thresholds. By “precision,” we mean how close the thresholds were to each other. By “robustness,” we mean how insensitive the thresholds were to different simulation conditions. In the conditions without RG, however, we examined the proportions of specific conditions in cases for which a threshold was estimated.

Finally, we addressed RQ3 by examining the average misclassification rates and proportions of false positives and false negatives. We computed the averages in groups broken by the RG component’s mixture weight and mean, because they were the only varied parameters that affected the population-level misclassification rates given by the optimal thresholds. In addition, the RG proportion determined the maximum values of the proportions of false negatives (PFN) and positives (PFP); the maximum PFN value was *λ*_*RG*_ and the maximum PFP value was $1-\lambda_{\mathrm{RG}}$ .

## Results

### Analysis of the Estimated Thresholds (RQ1)

**Figure 1.**
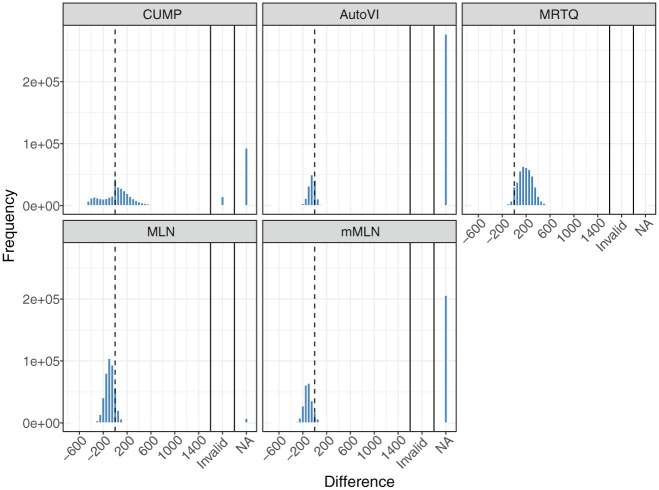
Distributions of the Differences Between the Estimated Thresholds Given by the Methods and the Optimal Thresholds Closest to Respective Method’s Thresholds in the Generated Samples in the Conditions with Rapid Guessing in the Population. *Note.* Invalid = the threshold was greater than the exponent of the log response time mode corresponding to the SB component (exp(7.5) ≈ 1,808); NA = not available, i.e., a threshold could not be estimated. Zero difference is indicated by the vertical, dashed lines.

Figure 1 shows the distributions of the differences between the thresholds estimated by the five methods and the time points at which the misclassification rates were minimized (“optimal” thresholds) that were closest to the respective method’s thresholds in the generated samples in the conditions where the population contained RG. We divided the x-axes such that cases for which the threshold was greater than the exponent of the log RT mode corresponding to the SB component ( $\exp\left(\mu_{\mathrm{SB}}\right)=\exp\left(7.5\right)\approx 1,808$ ), as well as missing values, were displayed as “invalid” and “NA” (not available), respectively. We considered thresholds greater than the exponent of the log RT mode to be invalid because these types of thresholds would likely not be used to detect RG in practice, as they would filter out too many responses and produce inaccurate results. In addition, Table 2 contains statistics of the estimated thresholds and their biases, calculated across valid cases, both in the conditions with and without RG. By “threshold bias,” we mean the average difference between the estimated thresholds and the optimal thresholds closest to the respective method’s thresholds. Note that the bias values in Table 2 in the conditions with RG are the means of the distributions shown in Figure 1.

**Table 2.** Statistics of the Estimated Thresholds and Their Biases Calculated Across Valid Cases Both in the Conditions With and Without Rapid Guessing (RG) in the Population.

	Conditions with RG				Conditions without RG		
Method	*Perc. est.*	*Mean*	*SD*	*Bias*	*Perc. est.*	*Mean*	*SD*
Optimal_S_	100	429	78	0	0	-	-
Optimal_L_	100	450	77	0	0	-	-
CUMP	75	483	279	39	13.6	785	260
AutoVI	35.8	374	98	-44	0	-	-
MRTQ	99.7	638	135	189	<0.1	800	292
MLN	98.2	340	37	-91	<0.1	1,096	497
mMLN	52.2	294	27	-113	<0.1	1,028	533

*Note*. *Optimal*_*S*_ = the smallest time points at which the misclassification rates were minimized in the generated samples; *Optimal*_*L*_ = the largest time points at which the misclassification rates were minimized; *Perc. est.* = percentage of cases for which a valid threshold was estimated; *SD* = standard deviation; *Bias* = mean of the difference between the estimated thresholds and the optimal thresholds closest to the respective method’s thresholds. The total number of cases was 100 × 4,320 = 432,000 in the conditions with RG and 100 × 360 = 36,000 without RG. Valid cases were thresholds smaller than the engaged component’s mean.

Figure 1 illustrates some interesting phenomena. First, CUMP was the only method to produce a bimodal distribution. However, the overall mode of the distribution is at zero, and, based on Table 2, CUMP had low bias, which indicates that the method was very accurate with the successfully estimated thresholds. However, CUMP was the only method to produce a notable number of invalid cases (3.4%). Excluding the invalid thresholds reduced the standard deviation (*SD*) of the CUMP thresholds from 2,284 to 279, indicating that they inflated the results substantially. Although the *SD* decreased, CUMP still exhibited the greatest threshold variation (i.e., the lowest precision). Finally, from Table 2, we see that only CUMP produced a notable number of thresholds in the conditions without RG. Before filtering out the invalid cases, the percentage of cases with a CUMP threshold was 16.9%.

Second, while AutoVI and mMLN produced substantial numbers of missing values, MRTQ and MLN produced relatively few. However, a closer look at the missing values showed that all of the missing values for MLN and mMLN occurred because of the bimodality remaining hidden in the estimated density of RT or log RT, respectively. Moreover, because 99.8% of the distributions were correctly judged to contain two components when assessing the number of components in the log RT distribution before estimating the AutoVI and the mixture model-based thresholds (see the “Method” section), all of this suggests that even when the two-component mixture distribution underlying the RTs was hidden from AutoVI in the observed RT distribution, the two-component mixture model could detect it in the observed log RT data. Nevertheless, based on Table 2, AutoVI was accurate, suggesting that whenever it was able to estimate a threshold, it did so well. In addition, MLN outperformed MRTQ based on accuracy and precision.

### Parameters Affecting the Results (RQ2)

Upon examining the distributions of the differences between the thresholds produced by the RTTMs and the optimal thresholds in the conditions with RG, two phenomena emerged that could not be fully explained: (1) the distribution of the CUMP differences overall (shape, invalid cases, and missing values) and (2) the large number of missing values in the AutoVI and mMLN results. In addition, the substantial number of CUMP thresholds in the conditions without RG prompted the question of whether some parameters contributed to this phenomenon more than others. To address these phenomena and RQ2, we examined the differences to the optimal thresholds when RG was present and the number of CUMP thresholds when RG was not present in more detail by accounting for the effects of the simulation parameters.

#### Parameters Affecting the AutoVI, MRTQ, MLN, and mMLN Results in the Conditions With RG

**Figure 2.**
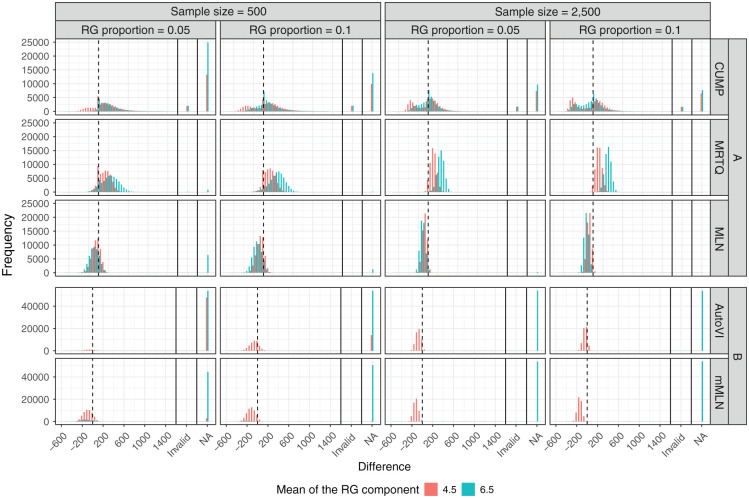
Distributions of the Differences Between the Estimated Thresholds Given by the Methods and the Optimal Thresholds Closest to the Respective Method’s Thresholds in the Generated Samples in the Conditions With Rapid Guessing in the Population, Broken by the Sample Size (*n*), the Proportion of Rapid Guesses (*λ*_*RG*_), and the Mean Log Response Time in the Rapid-Guessing Component (*μ*_*RG*_). *Note.* Invalid = the threshold was greater than the exponent of the log response time mode corresponding to the SB component (exp(7.5) ≈ 1,808); NA = not available, i.e., a threshold could not be estimated for this case. Zero difference is indicated by the vertical, dashed lines. The y-axis scales between plots A and B are different.

The phenomena regarding AutoVI and the MMAs in the situation with RG could be explained by considering the parameters that affected the shapes of the observed RT or log RT distributions: the sample size (*n*), the mixture weight of the RG component (*λ*_*RG*_), and the mode of the log RT distribution corresponding to the RG component (*μ*_*RG*_). The detailed results can also be seen visually in Figure 2, where the histograms of the differences to the optimal thresholds closest to the respective method’s thresholds are grouped by *n*, *λ*_*RG*_, and *μ*_*RG*_. Note that due to the large number of missing values in the AutoVI and mMLN results, which would have influenced the scales of the y-axes of the other methods’ histograms, Figure 2 is broken into plots A and B, where plot A contains the histograms of CUMP, MRTQ, and MLN, and plot B contains the histograms of AutoVI and mMLN.

Of these methods, AutoVI and mMLN were the only methods to produce substantial numbers of missing values. Regarding AutoVI, 77.7% of the missing values occurred when *μ*_*RG*_= 6.5, and 99.7% of the cases with *μ*_*RG*_= 6.5 were missing values. For *μ*_*RG*_= 4.5, a smaller sample size accounted entirely for the missing values, as 99.9% of them occurred when *n*= 500. The RG component’s mixture weight was also a contributing factor: 77.3% of these cases occurred when *λ*_*RG*_= 0.05, and combined with the sample size, 77.2% occurred when *n*= 500 and *λ*_*RG*_= 0.05. However, for *μ*_*RG*_= 6.5, there was no difference between sample sizes or mixture weights, as 25% of the missing values occurred under the four combinations of sample size and mixture weight. Thus, *μ*_*RG*_ was the main factor affecting the AutoVI thresholds, followed by *n*, and then *λ*_*RG*_. The supplementary material (available at: https://doi.org/10.17605/OSF.IO/VJNR7) contains a figure that illustrates two RT distributions from the simulations as example cases of situations in which AutoVI failed or succeeded in estimating an appropriate threshold. Regarding mMLN, the only factor contributing to the missing values was *μ*_*RG*_; 98.4% of the missing values occurred when *μ*_*RG*_= 6.5, and 94% of the cases with *μ*_*RG*_= 6.5 were missing values.

While the distribution produced by MRTQ initially appeared to be unimodal in Figure 1, bimodal shapes emerged when accounting for *n*, *λ*_*RG*_, and *μ*_*RG*_, though only *μ*_*RG*_ was necessary to observe the bimodality, as seen in Figure 2. Interestingly, when the sample size was smaller, especially in the cases with *μ*_*RG*_= 4.5 produced differences close to zero, as these differences appear to be over-represented compared to the others. Moreover, cases with *μ*_*RG*_= 6.5 appeared to have greater variation in the differences than cases with *μ*_*RG*_= 4.5, as the pooled variances across groups broken by *n* and *λ*_*RG*_ were 15,887 and 8,346; respectively. In addition, the sample size and the mixture weight contributed to the variance: 85.6% of the pooled variance of the cases with *μ*_*RG*_= 6.5 was accounted for when *n*= 500; 55.6% was accounted for when *λ*_*RG*_= 0.05; and 47.7% was accounted for when *n*= 500 and *λ*_*RG*_= 0.05.

For MLN, clear bimodal shapes did not appear when accounting for *n*, *λ*_*RG*_, and *μ*_*RG*_. In addition, there were no meaningful differences in the variation of the differences between cases with *μ*_*RG*_= 6.5 and cases with *μ*_*RG*_= 4.5, as the pooled variances were 5,310 and 4,090; respectively. Similar to MRTQ, however, the sample size affected the variance of the MLN differences, such that 78.2% of the pooled variance for cases with *μ*_*RG*_= 6.5 and 76.4% for cases with *μ*_*RG*_= 4.5 was accounted for by the smaller sample size. Moreover, cases with *μ*_*RG*_= 6.5 produced all of the missing values for MLN, and when accompanied by the smaller sample size, this phenomenon was more prominent. Finally, MRTQ and MLN differed from each other as cases in both groups of *μ*_*RG*_ produced mostly positive differences for MRTQ, but mostly negative differences for MLN.

#### Parameters Affecting the CUMP Results

*Invalid Cases and Missing Values in the Conditions with RG*. Invalid CUMP thresholds mostly occurred in cases where only a little change occurred in the cumulative proportion of correct responses as a function of RT (i.e., the CUMP curve) when moving from zero toward the maximum RT in the sample; 72.3% of the invalid thresholds were produced when the chance level (*g*) was 0.5, the item difficulty (*b*) was 0, and the pseudo-guessing (*c*) was 0. Moreover, 19.8% were produced when *g*=0.25, *b*= 1, and *c*= 0, summing up to 92.1%. In these situations, the CUMP curve often crossed the chance level line late in relation to RT. Thus, the CUMP method was prone to producing large thresholds. For instance, when *b*= 0 and *c*= 0, regardless of the value of the item discrimination parameter (*a*), the accuracy of engaged responses generated according to the 3PL IRT model was approximately equal to 0.5, which is the chance level for two-choice items. However, when *b*= 1 and *c*= 0, the resulting accuracy level depended on *a*; cases with *a*=1.5 produced accuracy levels closest to the chance level for four-choice items (*g*=0.25). By considering this parameter in addition to *b* and *c*, we found that 97.5% of the invalid cases with *g*=0.25, *b*= 1, and *c*= 0 occurred when *a*=1.5.

The sample size, the mean of the RG component, the mixture weight of the RG component, and the error in the rapid-guessing accuracy (*e*_*g*_) accounted for some of the cases in which a threshold could not be estimated. Specifically, 66.4% of missing values were produced when *n*= 500, 60.3% were produced when *μ*_*RG*_= 6.5, 59.3% were produced when *λ*_*RG*_= 0.05, and 20.1%, 29.7%, and 50.2% were produced when $e_{g}=-0.05$ , *e*_*g*_= 0, and $e_{g}=0.05$ , respectively. However, these parameters did not produce interacting effects, as the discrepancies in the group proportions for each parameter remained even when accounting for the other three parameters. For example, regardless of the sample size, the mean of the RG component, or the mixture weight of the RG component, the proportions of missing values in the *e*_*g*_ groups remained in the same order of magnitude as above. In addition, regardless of the value of *e*_*g*_, most missing values occurred when *n*= 500, *μ*_*RG*_= 6.5, and *λ*_*RG*_= 0.05. For the other parameters, i.e., the chance level (*g*), the item parameters in IRT (*a*, *b*, and *c*), and the correlation between ability and speed ( $\rho_{\theta \tau }$ ), the effect was not as strong, or there was no effect.

*Shape of the Distribution of the Differences Between the CUMP Thresholds and the Optimal Thresholds in the Conditions with RG*. When the sample size was smaller, cases with *μ*_*RG*_= 4.5 produced more conservative thresholds (negative differences) than cases with *μ*_*RG*_= 6.5 (63.1% vs. 36.9%), which was likely due to CUMP failing to estimate thresholds that would have been conservative for cases with *μ*_*RG*_= 6.5 (see Figure 2). However, based on Figure 2, when the sample size was larger, cases with *μ*_*RG*_= 4.5 produced more very conservative thresholds (e.g., negative differences below -200 ms). In addition, the error in the rapid-guessing accuracy explained both the negative and positive differences quite well; 21.4%, 36.9%, and 41.6% of the negative differences were produced when $e_{g}=-0.05$ , *e*_*g*_= 0, and $e_{g}=0.05$ , respectively, while 47.1%, 32.4%, and 20.5% of the positive differences were produced when $e_{g}=-0.05$ , *e*_*g*_= 0, and $e_{g}=0.05$ , respectively. These proportions remained in the same order of magnitude, regardless of the sample size. For the other parameters, the main effect was not as strong, or there was no effect. Moreover, similar to the MRTQ method, differences close to zero were over-represented, especially when the sample size was smaller (see Figure 2).

*Thresholds in the Conditions Without RG.* The CUMP method was the only one that produced a considerable number of thresholds when the population did not contain RG (13.6% of cases). Three parameters, *g*, *c*, and *b*, contributed to this phenomenon: 62.9% of all CUMP thresholds occurred when *g*=0.5, 73.7% occurred when *c*= 0, and 96.6% occurred when b≥0 . When considering only the cases with b≥0 , these parameters had an interacting effect: When *g*=0.25, mostly cases with *c*= 0 produced thresholds, with the number of thresholds increasing as *b* increased: 1.9% (*b*= 0), 28.3% (*b*= 1), and 47.6% (*b*= 2), summing up to 77.8%. Similar to above, when *g*=0.5, mostly cases with *c*= 0 produced thresholds. However, in contrast to the cases with *g*=0.25, the number of thresholds decreased as *b* increased: 36.8% (*b*= 0), 26.8% (*b*= 1), and 6.2% (*b*= 2), summing up to 69.8%. The supplementary material (available at: https://doi.org/10.17605/OSF.IO/VJNR7) contains a figure that illustrates six CUMP curves from the simulations.

### Misclassifications (RQ3)

Table 3 contains the averages of the misclassification rate (MR), the proportion of false positives (PFP), and the proportion of false negatives (PFN), grouped by the RG component’s mixture weight and log RT mean. The average MR, PFP, and PFN were calculated based on the valid thresholds when the RG was present in the population. The distributions of MR, PFP, and PFN are visualized in the supplementary material (available at: https://doi.org/10.17605/OSF.IO/VJNR7). In this simulation setting, false positives (FPs) are true engaged responses that are identified as rapid guesses, and false negatives (FNs) are true rapid guesses that are identified as engaged responses. Because a misclassification is interpreted as either a false negative or a false positive, MR equals the sum of PFP and PFN. Note that because AutoVI and mMLN estimated valid thresholds for only 0.3% and 8.9% of all cases with *λ*_*RG*_= 0.05 and *μ*_*RG*_= 6.5, respectively, as well as 0.3% and 3.2% of all cases with *λ*_*RG*_= 0.1 and *μ*_*RG*_= 6.5, respectively, the results of these methods for cases with *μ*_*RG*_= 6.5 are not meaningfully comparable to the optimal situations or the other RTTMs.

**Table 3.** Averages of the Misclassification Rate and the Proportions of False Positives and Negatives, Calculated Across Valid Cases in the Conditions With Rapid Guessing.

	*λ*_*RG*_ = 0.05				*λ*_*RG*_ = 0.1			
Method	*Perc. est.*	*MR*	*PFP*	*PFN*	*Perc. est.*	*MR*	*PFP*	*PFN*
*µ*_*RG*_ = 4.5								
Optimal_S_	100	.008	.001	.007	100	.015	.002	.013
Optimal_L_	100	.008	.001	.007	100	.015	.002	.013
CUMP	77.6	.029	.018	.011	81.5	.047	.021	.026
AutoVI	55.8	.011	.002	.010	87	.017	.001	.016
MRTQ	100	.013	.007	.006	100	.022	.011	.011
MLN	100	.010	0	.010	100	.018	.001	.017
mMLN	97.3	.011	0	.011	99.7	.021	0	.020
*µ*_*RG*_ = 6.5								
Optimal_S_	100	.031	.001	.030	100	.060	.004	.056
Optimal_L_	100	.031	.002	.029	100	.060	.005	.055
CUMP	64.4	.048	.019	.029	76.6	.078	.019	.059
AutoVI^a^	0.3	.344	.329	.015	0.3	.332	.303	.030
MRTQ	99.1	.051	.025	.025	99.9	.091	.045	.046
MLN	93.9	.035	0	.035	98.8	.067	.001	.067
mMLN^a^	8.9	.034	0	.034	3.2	.068	0	.067

*Note*. *Optimal*_*S*_ = the smallest time points at which the misclassification rates were minimized in the generated samples; *Optimal*_*L*_ = the largest time points at which the misclassification rates were minimized; *λ*_*RG*_ = rapid-guessing (RG) component’s mixture weight (i.e., the RG proportion); *µ*_*RG*_ = RG component’s log RT mean; *Perc. est.* = percentage of cases for which a valid threshold was estimated; *MR* = misclassification rate; *PFP* = proportion of false positives; *PFN* = proportion of false negatives. The displayed values are averages of the corresponding measure. The total number of cases was 100 × 4,320/4 = 108,000 for all four groups. Valid cases were thresholds smaller than the engaged component’s mean.

a These results are not meaningfully comparable to the other methods’ results because of the large discrepancy in the number of estimated thresholds.

Based on Table 3, the average minimum MR possible (i.e., the average MR produced by the optimal thresholds) increases as a function of *λ*_*RG*_ and *μ*_*RG*_, largely due to the average PFN value increasing, but PFP remaining constant, regardless of the selection of the optimal threshold. Consequently, the average MR values given by CUMP, MRTQ, and MLN increase as functions of these parameters. CUMP produced the largest average MR values for cases with *μ*_*RG*_= 4.5 and MRTQ for cases with *μ*_*RG*_= 6.5. For cases with *μ*_*RG*_= 4.5, the MMAs produced similar average MR values for both RG proportions. For cases with *μ*_*RG*_= 6.5, mMLN was not meaningfully comparable to MRTQ and MLN; however, between MRTQ and MLN, MLN produced the smaller average MR, regardless of the RG proportion. In fact, MLN’s average MR values were close to the optimal values, differing by only 0.007 at the largest when *λ*_*RG*_= 0.1 and *μ*_*RG*_= 6.5.

Most misclassifications produced by the optimal thresholds were FNs, as indicated by the average PFP values being smaller than the average PFN values. All RTTMs, except CUMP, for cases with *λ*_*RG*_= 0.05 and *μ*_*RG*_= 4.5, as well as MRTQ in general, produced this type of imbalance between FPs and FNs. Regardless of the group examined, MRTQ appeared to produce a balance between FPs and FNs, with PFP and PFN values equal or nearly equal, differing by only 0.001 at the largest. This phenomenon is not ad hoc, but also occurs at the population level due to the definition of the MRTQ threshold, as discussed in the supplementary material.

## Discussion

RG identification using RTTMs presents us with two major challenges: (1) the inevitability of misclassifications, and (2) the absence of agreement on the best method to use to identify RG under varying conditions. The current simulation study was designed to address these two challenges in an unbiased setting. We generated data according to either a one-component model, assuming the population did not contain RG, or a two-component mixture model, assuming the presence of RG in addition to SB, by varying several parameters that may have affected the misclassification rates produced by the RTTMs. The total number of simulation conditions was 4,680. We compared five RTTMs: the CUMP method, the AutoVI method, and three MMAs: MRTQ, MLN, and a modified version of MLN (mMLN). The results were interpreted separately for the conditions with and without RG, because the goal of RG identification is different between these two conditions: with RG, the optimal threshold is the time point that minimizes the misclassification rate, whereas without RG, the optimal threshold does not exist, and a threshold should not be estimated.

First, when the population contained RG, the results showed that MLN was the most accurate (low bias, i.e., small average difference to the optimal thresholds), robust (not sensitive to different conditions), and precise (low variability in the thresholds) of the RTTMs. The other methods had issues that hindered their accuracy, precision, and/or robustness. For instance, AutoVI and mMLN struggled with robustness, and CUMP struggled with robustness and precision. These results were consistent with those of Holopainen et al. (2025), who found that CUMP was the least robust method. However, in their study, MRTQ produced the lowest misclassification rates.

When the population did not contain RG, CUMP was the only method to estimate a significant number of thresholds. However, in practical settings, although most of the thresholds were valid in relation to the RT distribution, some practitioners would probably reject these thresholds, because of the absence of bimodality in the distribution, as RG and SB are usually conceptualized as two distinct distributions (e.g., Lee & Jia, 2014; Schnipke, 1995; Schnipke & Scrams, 1997; Wise, 2019). Furthermore, some practitioners would probably reject a CUMP threshold if the CUMP curve does not vary around the chance level at small RTs, which is characterized by a “zigzag” pattern.

Furthermore, we investigated which simulation parameters affected the results of which methods, and how. The AutoVI method and the MMAs were affected by the shape of the observed RT distribution in different ways. AutoVI produced missing values when it failed to detect bimodality, and mMLN had difficulty reflecting the observed bimodality in the estimated log RT density. Consequently, mMLN did not produce meaningful thresholds for nearly all cases with the larger log RT mean of the RG component (6.5). In contrast, MRTQ produced greater variation in the thresholds when bimodality was more difficult to detect. However, the MLN method again showed its robustness, as the variation in its thresholds changed the least. All in all, the most difficult condition for these methods was the smaller sample size (500), accompanied by the smaller RG proportion (0.05) and the larger mean of the RG component’s log RT distribution (6.5). In this condition, AutoVI failed to estimate a threshold in nearly all cases, MLN produced most of its missing values, and MRTQ produced the largest variation between the thresholds.

For CUMP, the interpretations were more complicated. First, in the conditions with RG, most invalid thresholds, i.e., the thresholds greater than the exponent of the log RT mode of the engaged responses, were estimated in cases where only a little change occurred in the CUMP curve. In other words, in these cases, the accuracy levels of rapid guesses and engaged responses were similar. However, most of these cases were two-choice test items with a lower asymptote at zero, which are atypical in educational testing. This type of item is likely a trick question or has something in the wrong answer option that attracts low-ability individuals to choose it over purely guessing, resulting in accuracies below the theoretical chance level of 0.5.

In contrast, the other type of items, which contributed to the invalid thresholds, i.e., the four-choice items with a lower asymptote at zero, are more common. For example, in the PISA assessments, which administer multiple-choice and open-response items, binary item responses are modeled and scaled using a unidimensional, multiple-group IRT model based on the two-parameter logistic model, where the lower asymptote is fixed at zero (OECD, 2024). Nevertheless, if the rapid-guessing and engaged accuracy are similar, this phenomenon may also occur for other types of items not examined in this study. This also depends on the dependency structure between ability and speed, because it affects the behavior of the CUMP curve; in this study, where we considered only positive correlations, the CUMP curve tended to first increase above the overall accuracy level after varying around the rapid-guessing accuracy in the sample, and then converge toward the overall accuracy, because the high-ability participants responded faster than the low-ability participants.

Second, many parameters accounted for some of the cases for which a threshold could not be estimated, with the most important factors being the sample size and the error in the rapid-guessing accuracy. Similar to the AutoVI method and the MMAs, the most difficult condition for CUMP was the smaller sample size (500) combined with the smaller RG proportion (0.05) and the larger log RT mean of the RG component (6.5), as most of the missing values occurred in this condition. It appears that although one of CUMP’s strengths is that it can be used even when the observed RT distribution is not bimodal, it is still heavily affected by the distribution’s characteristics, especially when the sample size is at least moderately small (e.g., 500) and the proportion of rapid guesses is low (e.g., 0.05). In addition, CUMP is prone to failing when the theoretical chance level does not match the observed chance level, because in this case, the CUMP curve tends to remain above or below the chance level line throughout the entire RT range.

Third, when the sample size was larger, cases with the smaller log RT mean of the RG component produced more large negative differences. This effect was likely due to the definition of the CUMP threshold, because the CUMP curve has less noise at very small RTs when the sample size increases and/or the RG component’s mean approaches zero, and thus, the CUMP threshold approaches zero. In addition, at the population level, because of the two-component mixture model for responses, the CUMP curve never crosses the chance level line within the region of the RG component, as the SB component influences the entire RT distribution even at small RTs close to zero.

Finally, similar to the invalid thresholds in the conditions with RG, the CUMP thresholds in the conditions without RG were mostly produced by items with a lower asymptote at zero, regardless of the chance level. In this case, however, the item difficulty parameter had a larger role, though thresholds were estimated mostly for items of medium difficulty or higher, that is, items with non-negative difficulty parameter values. When the lower asymptote is at zero, and the item is difficult, accompanied by positive correlations between ability and speed, the CUMP curve may be a decreasing function of RT at small RTs that starts above the chance level and inevitably crosses the chance level at some time point, which is then chosen as the CUMP threshold if not treated with caution. In real-world settings, this phenomenon would likely be due to high-ability participants answering faster than low-ability participants, who respond with lower accuracy than chance, meaning that response accuracy would be large at small RTs but decrease as RT increased.

We also investigated the misclassification rates produced by the methods. The superiority of MLN was supported by its average MR value being the smallest. The CUMP method produced the largest average MR value for cases with the smaller log RT mean of the RG component (4.5) and MRTQ for cases with the larger mean (6.5). This may be explained by the greater variance in the thresholds produced by CUMP and MRTQ compared to the other methods, which meant that they produced more thresholds close to the lower and upper bounds of the valid threshold range (from 0 to exp(7.5) ≈ 1,808). Excluding MRTQ, the methods seemed more conservative than liberal because they produced more false negatives than false positives. MRTQ produced an equal number of false negatives and false positives, as it should. These results were consistent with Wise (2017), Rios and Deng (2021), and Holopainen et al. (2025), who also found that most RTTMs are more conservative than liberal.

### Limitations and Future Research

There are important limitations to this study that must be addressed. Starting with method-specific limitations, we used the rule-of-thumb bandwidth proposed by Silverman (1981) to estimate kernel densities of RTs and find the AutoVI thresholds. Other bandwidths or the original visual inspection method (Schnipke, 1995) may have detected bimodality in situations where AutoVI failed. A more sophisticated approach would have first found the smallest bandwidth for the kernel density to have at most two modes (see, e.g., Ameijeiras-Alonso et al., 2019) and then used that to estimate the kernel densities of RTs. In addition, during data analysis, we found that the CUMP method could be improved in situations where rapid-guessing and engaged response accuracies were close by identifying the time point at which the CUMP curve first crossed the chance level line and remained above it for several time segments instead of finding the maximum RT at which the CUMP curve was less than or greater than the chance level (see the supplementary material available at: https://doi.org/10.17605/OSF.IO/VJNR7). A simple way to do this would have been to set the upper limit of the search space for a CUMP threshold to the overall RT mode or median RT. Improved versions of AutoVI and CUMP could be examined in future research.

There were also limitations regarding the model used to generate the data. First, the RTs we generated were much smaller than those found in real-world, large-scale assessments such as PISA in 2022. In the latter, the median RTs of mathematics items, calculated across skill levels, ranged from 0.6 to 2.6 minutes (OECD, 2024). Furthermore, Guo et al. (2016) validated their CUMP method using a sample collected with items for which the CUMP threshold ranged from 2 to 31 seconds. Most previous RG research has focused on items requiring more time to solve, presumably with greater cognitive demand than the hypothetical items in this study (e.g., Avvisati et al., 2024; Guo et al., 2016; Skalka & Valko, 2024; Wise, 2006, 2019; Wise & Kong, 2005; Wise & Ma, 2012).

Second, it was reasonable to interpret the two possible locations of the mode of the RG component as representing the two types of rapid guessing in low-stakes settings: total thoughtless rapid guessing (*μ*_*RG*_= 4.5) and rapid guessing by test-takers who quickly glanced at the item before deciding to guess (*μ*_*RG*_= 6.5). However, we did not consider that both types of rapid guessing may be present in the population. Third, based on earlier research (e.g., Klein Entink et al., 2009; Meng et al., 2015; Nagy & Ulitzsch, 2022; Ulitzsch et al., 2020; van der Linden, 2007; Wang & Xu, 2015), we assumed normal distributions for the abilities and speeds of engaged participants and the log RTs of both engaged and disengaged participants. However, this assumption may be violated in some situations. For instance, the ability distribution could include subpopulations of engaged individuals who use different strategies (e.g., AlHakmani & Sheng, 2024; Mislevy & Verhelst, 1990), or it could be skewed in either direction due to tests that are extremely easy or extremely difficult. In addition, other than the lognormal model for RT have been proposed in previous literature, such as the Weibull model (Rouder et al., 2003) or semi-parametric models (Wang et al., 2013). Fourth, we considered only positive correlations between ability and speed, but negative correlations may also occur in large-scale assessments (see, e.g., Goldhammer & Klein Entink, 2011; Goldhammer et al., 2014; Klein Entink et al., 2009). In sum, future research should consider a more comprehensive model with a wider range of possible RT modes and standard deviations, non-normal distributions for ability and speed, other models than the lognormal for RT, the two types of rapid guessing simultaneously, and negative correlations between ability and speed.

Fifth, in theory, such engaged responses might have been generated, for which the RT indicated a disengaged response. Holopainen et al. (2025) hypothesized that an engaged test-taker’s choice reaction time would reflect their absolute minimum response time to an item in an achievement test. For instance, Deary et al. (2011) found that the mean reaction time of young adults to the Deary-Liewald choice reaction time test was 474.5 milliseconds. Thus, if we had used 500 milliseconds as a rough estimate for the hypothetical test-takers’ choice reaction times in our setting, the probability of an engaged RT being less than 500 milliseconds would have been $P\left(L\leq \log\left(500\right);\mu_{\mathrm{SB}}=7.5,\sigma_{\mathrm{SB}}^{2}=0.5^{2}\right)\approx 0.005$ , indicating that it was nevertheless unlikely that these types of responses were generated.

The minor limitation discussed above was connected to the potential fallacy that we considered it a failure if a method estimated a threshold when the population did not contain RG; that is, was it a failure even if the estimated threshold was so small that responses with RTs below it must have been disengaged? However, interpreting responses with RTs below a certain threshold to be rapid guesses, even though the population was assumed to contain engaged responses only, presents a logical fallacy. Another question is whether it was truly a failure if the estimated threshold was smaller than the smallest RT in the generated sample, because in this case, the sample would be deemed to contain engaged responses only. However, only the AutoVI, MLN, and mMLN methods could have estimated such thresholds (see the supplementary material for the threshold definitions), but they produced zero or only a marginal number of thresholds in the conditions without RG in the first place. Therefore, we argue that this fallacy was more of a problem with the distribution from which the engaged log RTs were generated than our interpretation of a failure.

Similarly, such rapid guesses might have been generated for which the RT indicated an engaged response. For instance, the probability of a rapid-guessing log RT being greater than the mean of the SB component was $P\left(L>7.5;\mu_{\mathrm{RG}}=4.5,\sigma_{\mathrm{RG}}^{2}=1.5^{2}\right)\approx 0.023$ or $P\left(L>7.5;\mu_{\mathrm{RG}}=6.5,\sigma_{\mathrm{RG}}^{2}=1.5^{2}\right)\approx 0.252$ , indicating that a considerable proportion of such responses were generated, especially for cases with *μ*_*RG*_= 6.5. This prompts the question of whether responses with such RTs would be rapid guesses in reality. That is, could some rapid guessers be slower to respond than the engaged respondents on average? It could be argued that such responses do not represent rapid guessing but partial engagement (Wise & Kuhfeld, 2021). However, partially engaged responses are probably distributed differently from rapid guesses, and thus, they should not be generated from the same distribution. Nevertheless, future simulation studies of rapid guessing could mitigate this problem by carefully selecting an appropriate log RT variance for the RG component. For instance, if *σ*_*RG*_= 0.5, then $P\left(L>7.5;\mu_{\mathrm{RG}}=4.5,\sigma_{\mathrm{RG}}^{2}=0.5^{2}\right)<0.001$ and $P\left(L>7.5;\mu_{\mathrm{RG}}=6.5,\sigma_{\mathrm{RG}}^{2}=0.5^{2}\right)\approx 0.023$ .

Furthermore, although we found that the RTTMs were more conservative than liberal, the optimal thresholds were also conservative, implying that if the goal of threshold estimation in a practical setting with this type of data were to find the time point that minimizes MR, we would inevitably estimate conservative thresholds. It remains to be seen whether some other parameter values produce distributions for which the optimal threshold is actually more liberal than conservative. The results indicated that the balance between FPs and FNs given by the optimal thresholds became more even when either the mixture weight or the log RT mean of the RG component, or both, decreased. However, this was only because the average PFN decreased, but the average PFP remained stable. A more interesting case would be if the number of FPs grew. Presumably, if the log RT variance in the RG component was smaller than the SB variance and the components’ means were relatively close to each other, the proportion of engaged responses would be larger than the proportion of rapid guesses in the region where the RG and SB distributions overlap and also where the optimal threshold is located. Consequently, the number of FPs would exceed the number of FNs. Future research should confirm this hypothesis.

However, it is another question altogether whether a situation where the log RT variance of rapid guesses is smaller than the variance of engaged responses is realistic. In this study, the rapid-guessing variance was larger than the engaged variance, both of which were estimated from real, large-scale assessment data. Previous research has also found larger rapid-guessing log RT variance than engaged variance in real data (e.g., Nagy & Ulitzsch, 2022; Schnipke & Scrams, 1997; Ulitzsch et al., 2020). However, the order of magnitude between RG and SB variances depends on whether the original RT or log-transformed RT is examined; in our study, because the RTs were lognormally distributed, the RT variance of the rapid guesses with the smaller log RT mean was $(\exp\left(\sigma_{\mathrm{RG}}^{2}\right)-1)\exp\left(2\mu_{\mathrm{RG}}+\sigma_{\mathrm{RG}}^{2}\right)=(\exp\left(1.5^{2}\right)-1)\exp\left(2\times 4.5+1.5^{2}\right)=652,536.2$ , which was smaller than the RT variance of the engaged responses, i.e., $(\exp\left(\sigma_{\mathrm{SB}}^{2}\right)-1)\exp\left(2\mu_{\mathrm{SB}}+\sigma_{\mathrm{SB}}^{2}\right)=(\exp\left(0.5^{2}\right)-1)\exp\left(2\times 7.5+0.5^{2}\right)=1,192,197$ . Nevertheless, this phenomenon requires more research.

Finally, we generated data item by item, meaning that the hypothetical items were not linked. Consequently, we did not take into account the individuals’ tendencies of being engaged, nor the degree to which the items elicit disengagement (e.g., Ulitzsch et al., 2020). Future studies could consider this additional parameterization when examining misclassifications. In addition, due to item-wise data generation, we could not investigate the common k-time threshold method (Wise et al., 2004) or the change in information (ChInf; Wise, 2019) and change in information and accuracy methods (ChIA; Wise, 2019), nor the random search (RS) and genetic algorithm-based (GA) approaches (Bulut et al., 2023). All of these methods require data from entire tests with multiple items. However, examining the ChInf and ChIA methods or the RS and GA approaches in a simulation setting with large numbers of different conditions may not be practical anyway, because ChInf and ChIA require manually drawing and inspecting plots of item-total correlations, one item at a time (Wise, 2019), and the RS and GA approaches are computationally demanding (Bulut et al., 2023).

### Implications

Despite the limitations, our study had important implications for measurement practitioners. First, the results of AutoVI imply that it is prone to failing if the modes of the RG and SB components of the overall log RT distribution are too close to each other or if the sample size is small, especially when combined with a low proportion of rapid guesses. In practical settings, if there is a reasonable number of test items, one can visually inspect the item-specific RT distributions to determine if AutoVI is suitable, item by item, as is done with the original visual inspection method.

The results of the MMAs suggest these methods can be used as long as the population log RT distribution is bimodal, though a smaller sample size and a lower proportion of rapid guesses may reduce the precision of the estimates. In practical settings, this means that these methods can still be used even if the observed RT distribution does not appear to be bimodal based on visual inspection. Furthermore, CUMP can be used safely as long as its requirements are met: the observed rapid-guessing accuracy matches the theoretical chance level, and a sizable change in accuracy occurs as a function of RT when moving from small to large RTs. However, one should be aware of the possibility of low precision in CUMP thresholds across test items with varying characteristics, even when the requirements are met, as well as the possibility of CUMP providing thresholds in conditions where it should not be estimated.

In general, we recommend the following six-step process for RG identification at the item level:

1. Fit one- and two-component mixture models to the log RT data. Find evidence of bimodality:
    1.1. Compare the models’ fits to the data using information criteria (e.g., BIC) or the likelihood ratio test, for example. If the two-component mixture model better fits the data, then the distribution may be bimodal.
    1.2. Examine the estimated mixture weights and component means of the two-component model. If neither mixture weight is close to the average RG proportion of 10% found in low-stakes assessments (e.g., Rios et al., 2022)—for example, between 0% and 20%—or if one weight is close to this proportion but not linked to the smaller component mean, then the observed bimodality may not be due to RG, but rather to artificial modes in the tails of the distribution or other reasons. This would mean that the estimated log RT density cannot be meaningfully used to estimate an RG threshold.
    1.3. If there was evidence for bimodality in steps 1.1 and 1.2, proceed to step 2. Otherwise, skip steps 2 and 3 and proceed to step 4.
2. Estimate a threshold using a mixture modeling-based approach, such as MLN or MRTQ. If the goal is to minimize misclassification rate and/or find a more conservative than liberal threshold, we recommend the MLN method. If the goal is to find a more liberal threshold and/or a balance between the number of FPs and FNs, we recommend the MRTQ method.
3. Check the validity of the mixture modeling-based threshold. If a threshold could not be estimated or appears too large (e.g., greater than the overall RT mode or RT median), discard it and proceed to step 4. Otherwise, skip steps 4 and 5 and proceed to step 6.
4. Estimate a threshold using the CUMP method.
5. Check the validity of the CUMP threshold. If a threshold could not be estimated or appears too large, discard it. In addition, if the CUMP curve did not vary around the chance level at small RTs, treat the threshold with caution. This can be checked by, for example, calculating the mean accuracy of responses with RTs that characterize way too fast responding, and comparing this value to the chance level.
6. If a valid threshold was estimated in steps 1 to 5, use it for RG identification.

However, this process may be too complex for computerized adaptive testing settings, where test items are drawn from large pools of thousands of items. For these settings, simpler methods developed for this purpose are preferable, such as a common threshold for all items (Wise et al., 2004) or the normative threshold method with 10% (Wise & Ma, 2012) or 30% (Wise & Kuhfeld, 2021) thresholds. Nevertheless, practitioners should be mindful of the possibility of misclassifications that could have a negative impact on RG identification.
